# Supplementary material for: Robust thermometry-imaging down to sub-micrometer or millisecond-resolution by fluorescence lifetime microscopy allows for additional acquisition of multiple imaging channels
Source: Sci Rep. 2026 Aug 3;16:23768. doi: 10.1038/s41598-026-63244-1 (PMC13434772; doi:10.1038/s41598-026-63244-1)
Supplement: Supplementary file 1 [file 41598_2026_63244_MOESM2_ESM.pdf]

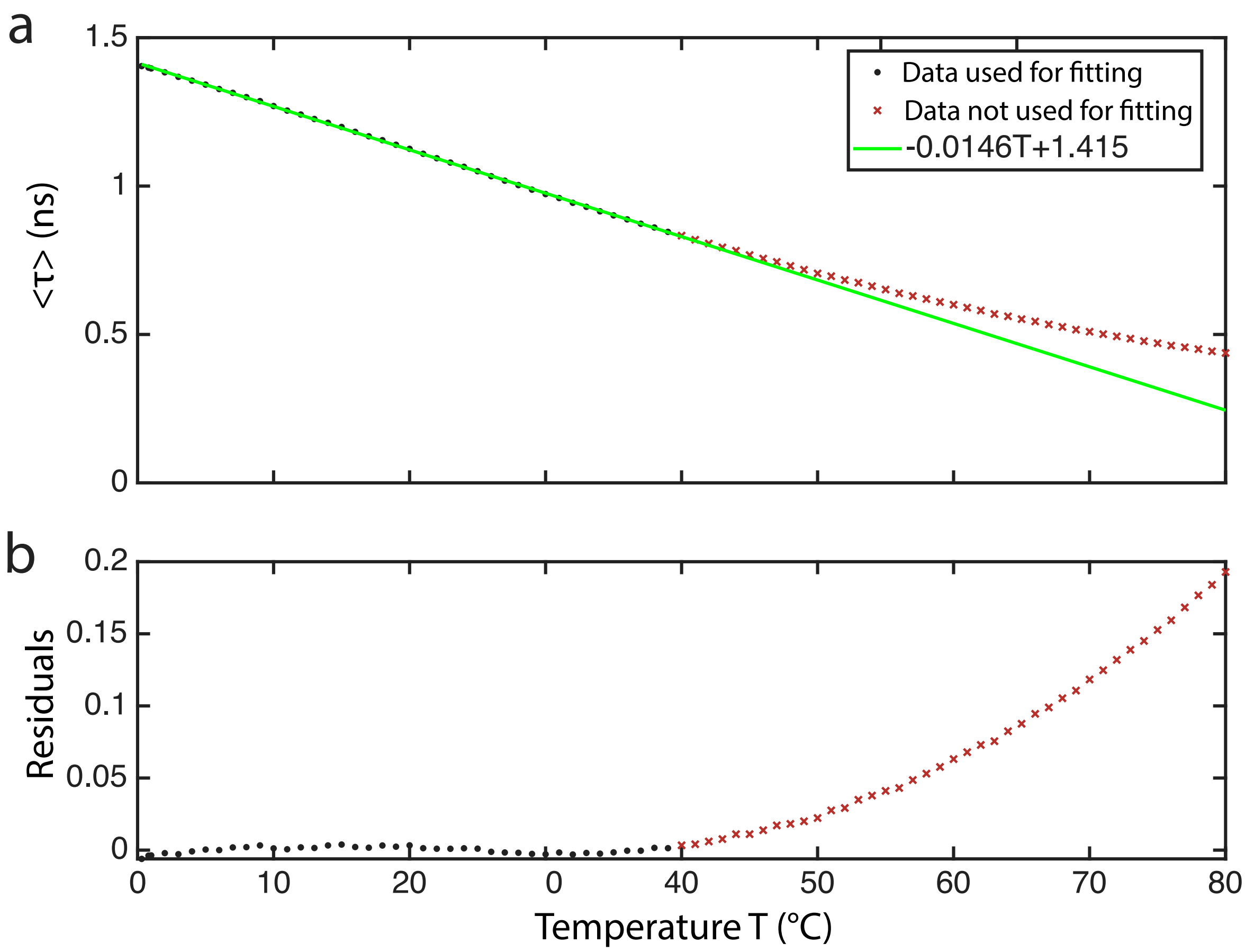

Supplementary Figure 1: Linear regression to fluorescence lifetime of Cy5 vs temperature
a) Plot of mean excited state lifetime (τ) of Cy5 in water (black dots and red crosses) measured at an Abberior Expert line confocal laser scanning microscope against temperature (N=4) together with a linear function (green line) fitted to the data range from 0-39°C (black dots) b) Residuals from the temperature region included (black dots) and excluded (red crosses) for linear fitting.

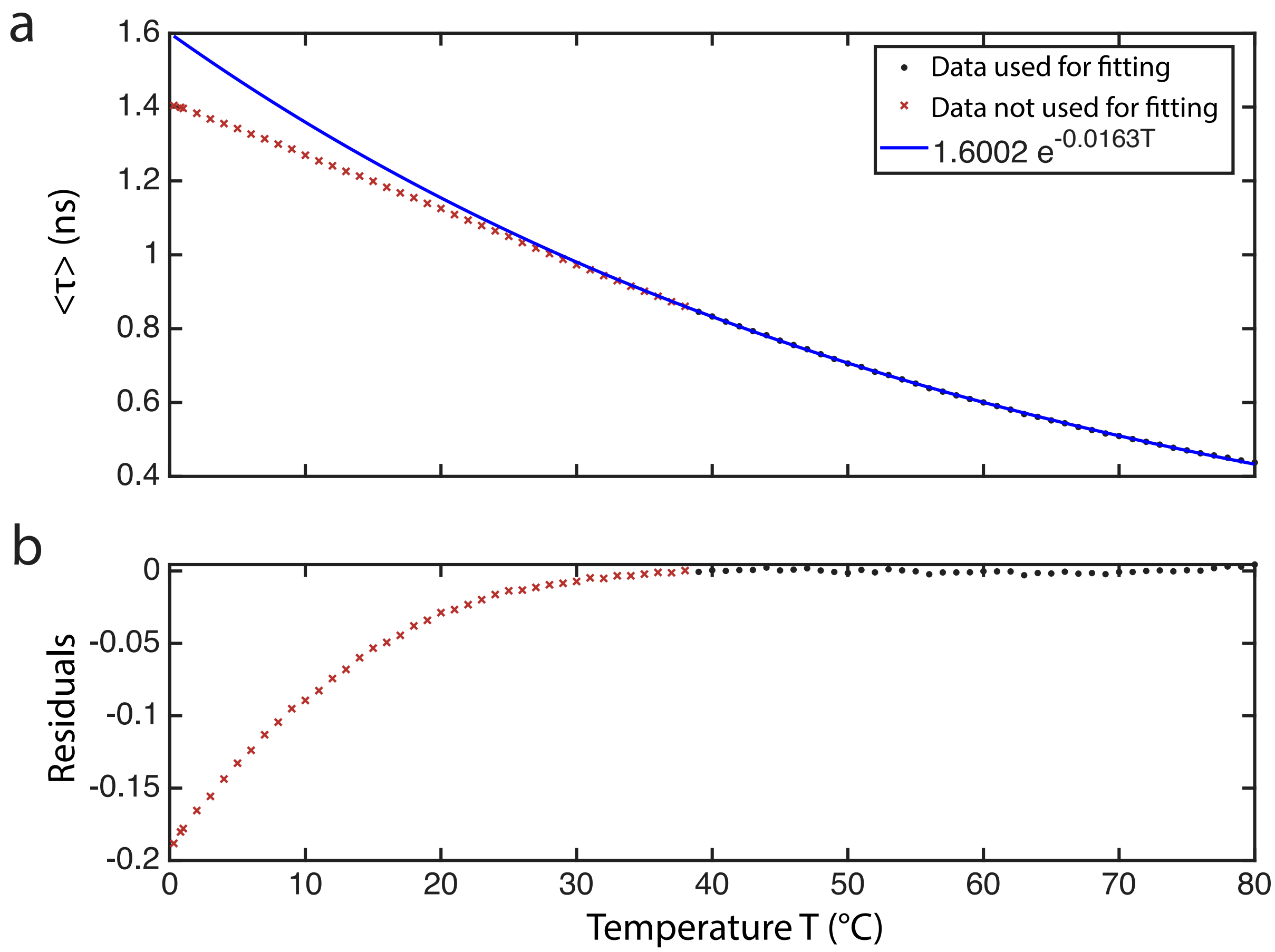

Supplementary Figure 2: Monoexponential regression to fluorescence lifetime of Cy5 vs temperature
a) Plot of mean excited state lifetime (τ) of Cy5 in water (red crosses and black dots) measured at an Abberior Expert line confocal laser scanning microscope against temperature (N=4) together with a monoexponential decay function fitted (blue line) to the data range from 40-80°C (black dots) b) Residuals from the temperature region included (black symbols) and excluded (red symbols) for fitting with a monoexponential function.

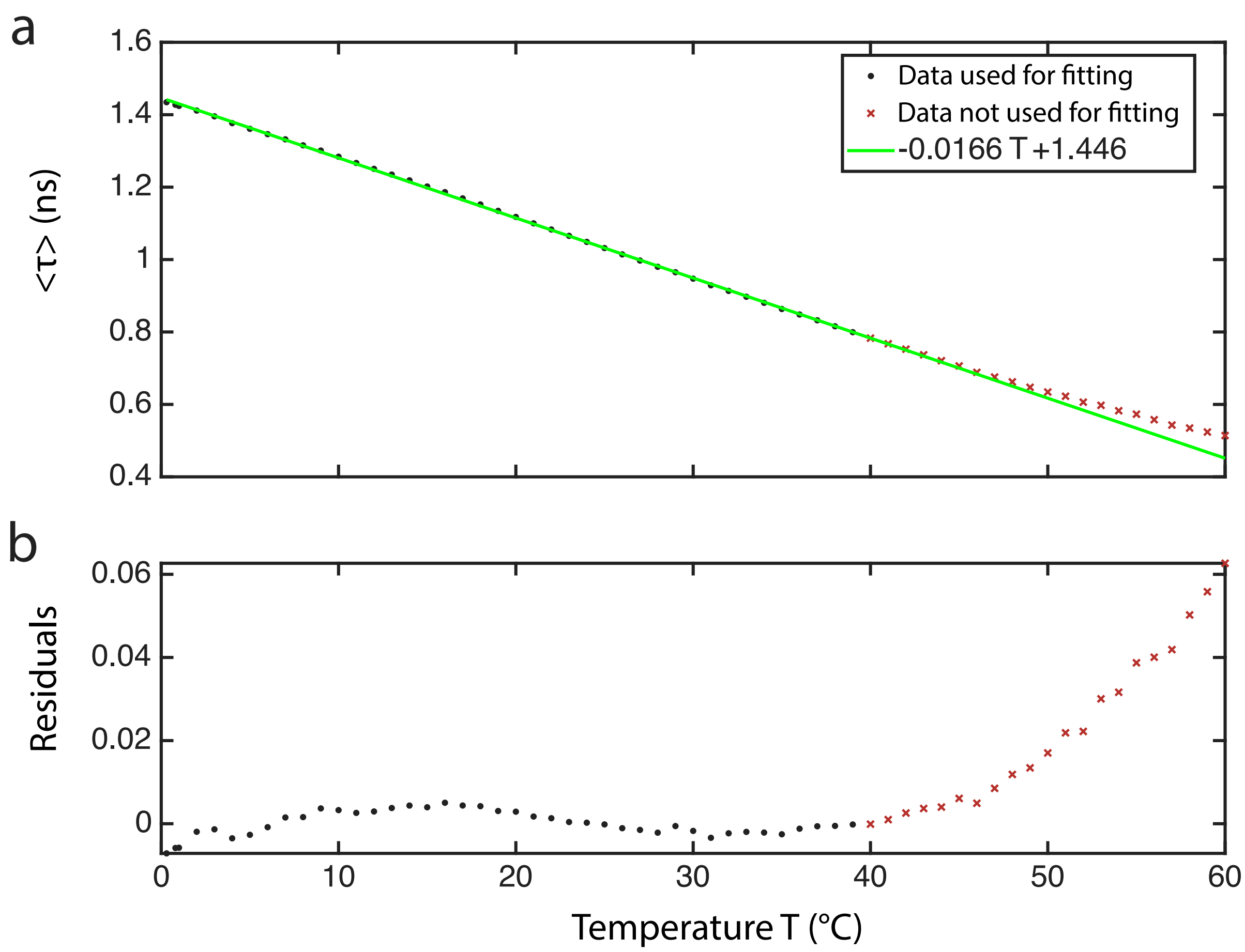

Supplementary Figure 3: Linear regression to fluorescence lifetime of Cy5 vs temperature (different microscope)

a) Plot of mean excited state lifetime (τ) of Cy5 in cell culture medium (black dots and red crosses) measured at a Leica SP8 confocal laser scanning microscope against temperature (N=4) together with a linear function fitted (green line) to the data range from 0-39°C (black dots) b) Residuals from the temperature region included (black dots) and excluded (red crosses) for linear fitting.

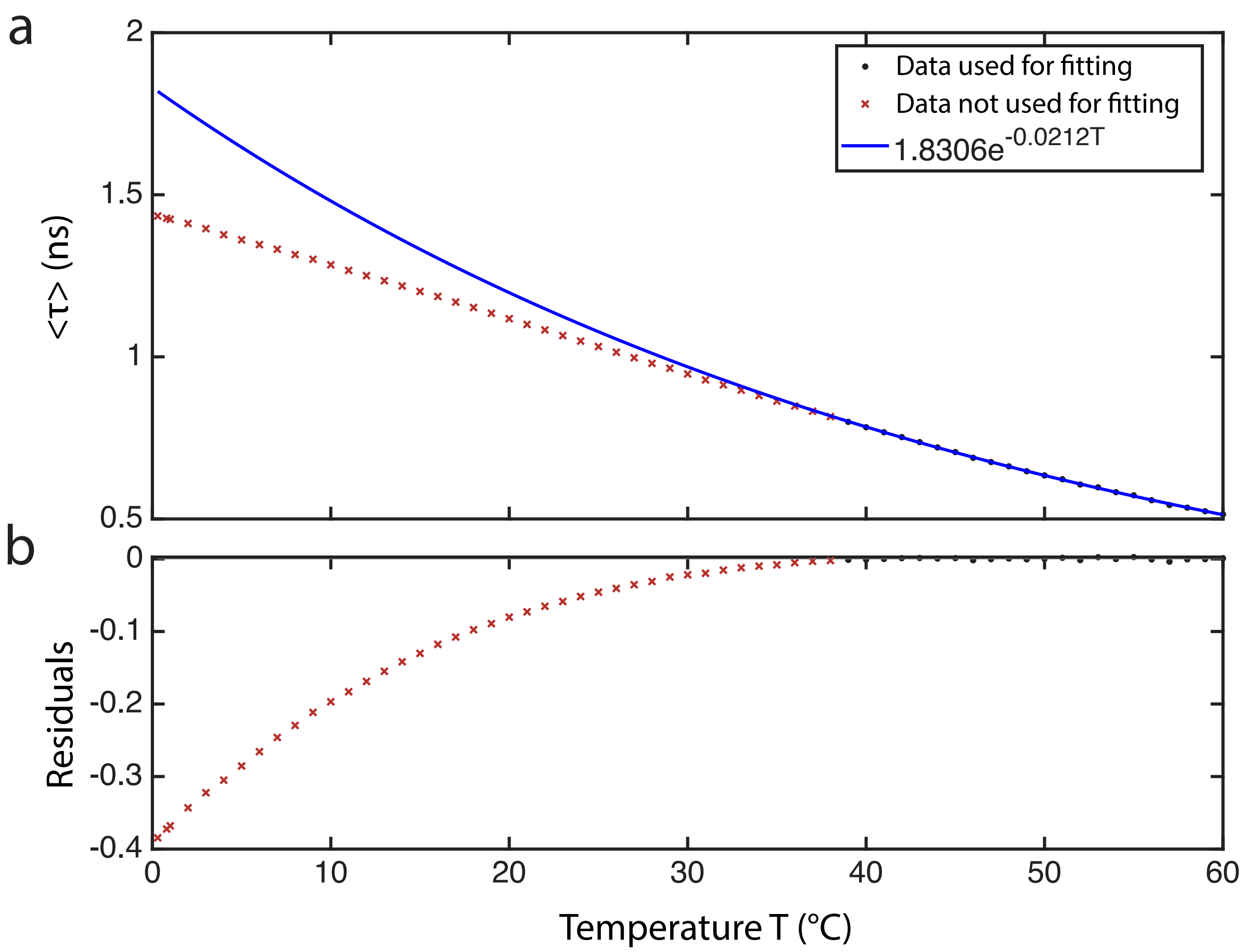

Supplementary Figure 4: Monoexponential regression to fluorescence lifetime of Cy5 vs temperature (different microscope)

a) Plot of mean excited state lifetime (τ) of Cy5 in cell culture media (black dots and red crosses) measured at a Leica SP8 confocal laser scanning microscope against temperature (N=4) together with a monoexponential decay function fitted (blue line) to the data range from 40-60°C (black dots) b) Residuals from the temperature region included (black dots) and excluded (red crosses) for fitting with a monoexponential function.

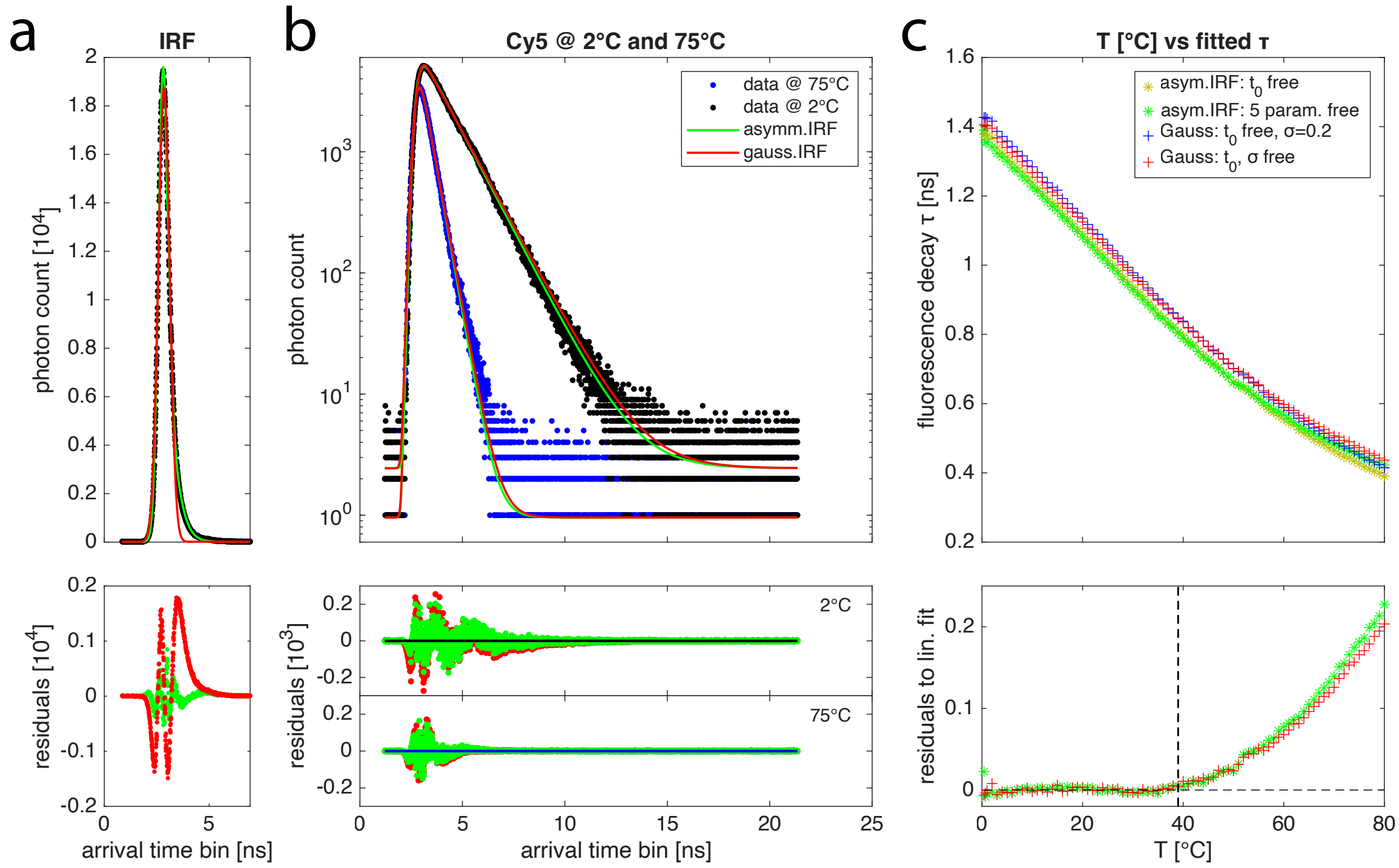

Supplementary Figure 5: Impact of different IRF-models with parameters inferred from arrival-time histogram a)-c): Top: measurement with fits, Bottom: respective residuals between data and respective fit. a) IRF measured as the histogram of number of photons of reflected pulsed 640-nm laser excitation (black dots) together with Gaussian fit (red line) and 5-parameter gaussian with asymmetric flanks (green line). b) Fit of a monoexponential fluorescence decay of Cy5-photon-arrival-times at 75°C (blue dots) and 2°C (black dots) individually convoluted with the two IRF-models in (a). c) Plot of fitted arrival times τ against externally controlled temperature T. Red: Fit with gaussian IRF described by parameters t_0 (center of Gaussian) and σ (width of Gaussian), free for each T; blue: using fixed σ=0.2 ns and free t_0 to fit the data; green: Fit with asymmetric IRF-model and individual 5 IRF-related parameters (t_0, and 4 parameters defining σ and asymmetric flanks) for each T; yellow: same as green, but fixing the 4 parameters defining σ and asymmetric flanks to those obtained at 39°C.

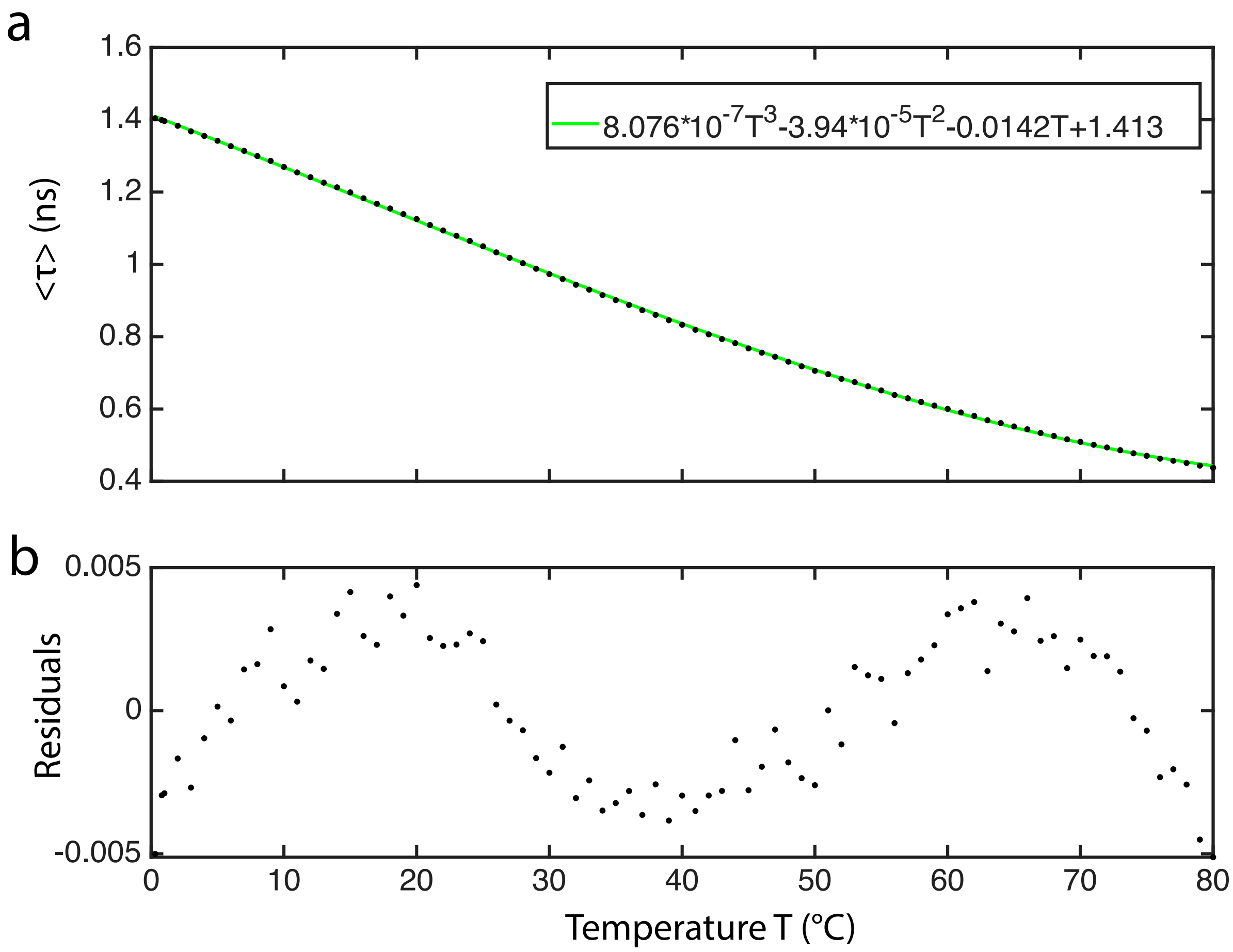

Supplementary Figure 6: Cubic regression to fluorescence lifetime of Cy5 vs temperature

a) Plot of mean excited state lifetime (τ) of Cy5 in cell culture media (black dots) measured at an Abberior Expert line confocal laser scanning microscope against temperature (N=4) together with a cubic fit of the data (green line) b) Residuals from the fit (black dots).

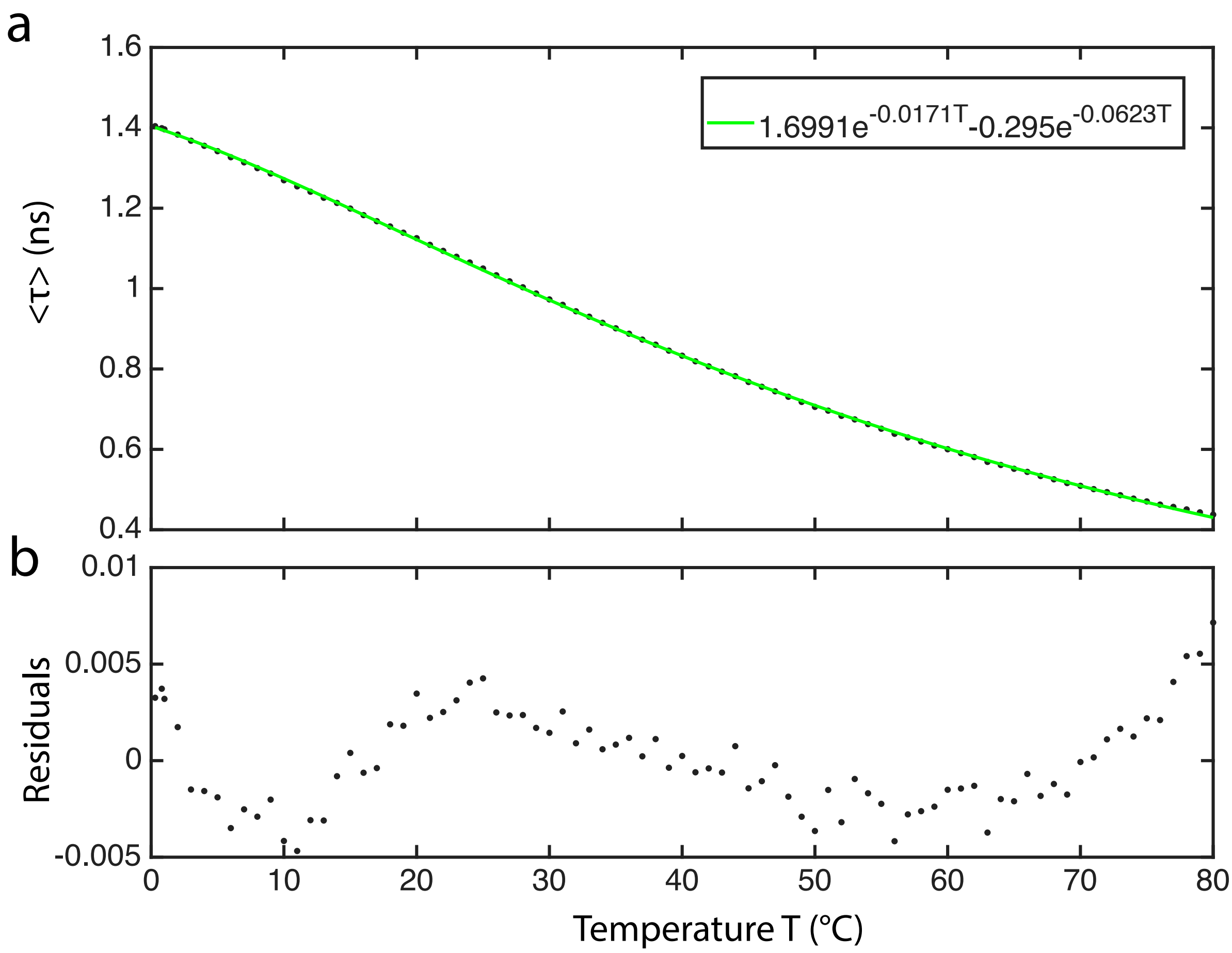

Supplementary Figure 7: Biexponential regression to fluorescence lifetime of Cy5 vs temperature
a) Plot of mean excited state lifetime (τ) of Cy5 in cell culture media (black dots) measured at an Abberior Expert line confocal laser scanning microscope against temperature (N=4) together with a biexponential fit of the data (green line) b) Residuals from the fit (black dots).

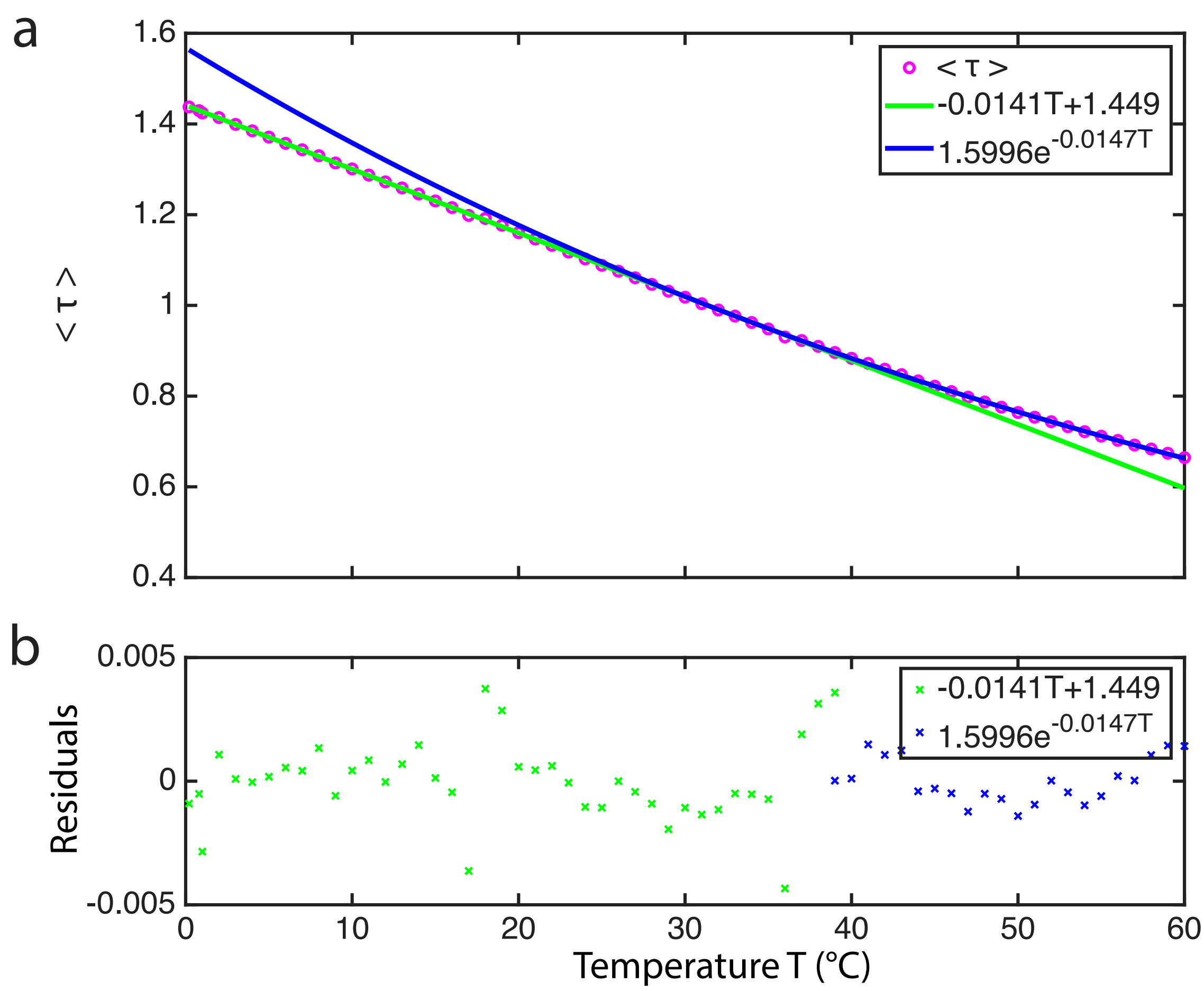

Supplementary Figure 8: Excited state lifetime of Cy5 in cell culture buffer as function of temperature

a) Excited state lifetime of Cy5 in a cell culture buffer (DMEM with 10% FCS) was measured over the temperature range 0-60°C on an Abberior Expert line CLSM. Shown is the mean excited-state lifetime (τ) of Cy5 (circles) over temperature from N=3 independent experiments together with a linear function fitted to the data range from 0-39°C (green line) and a monoexponential function fitted to the temperature range 40°C-60°C (blue line). b) Residuals in both fit-regimes: linear (green crosses), monoexponential (blue crosses).

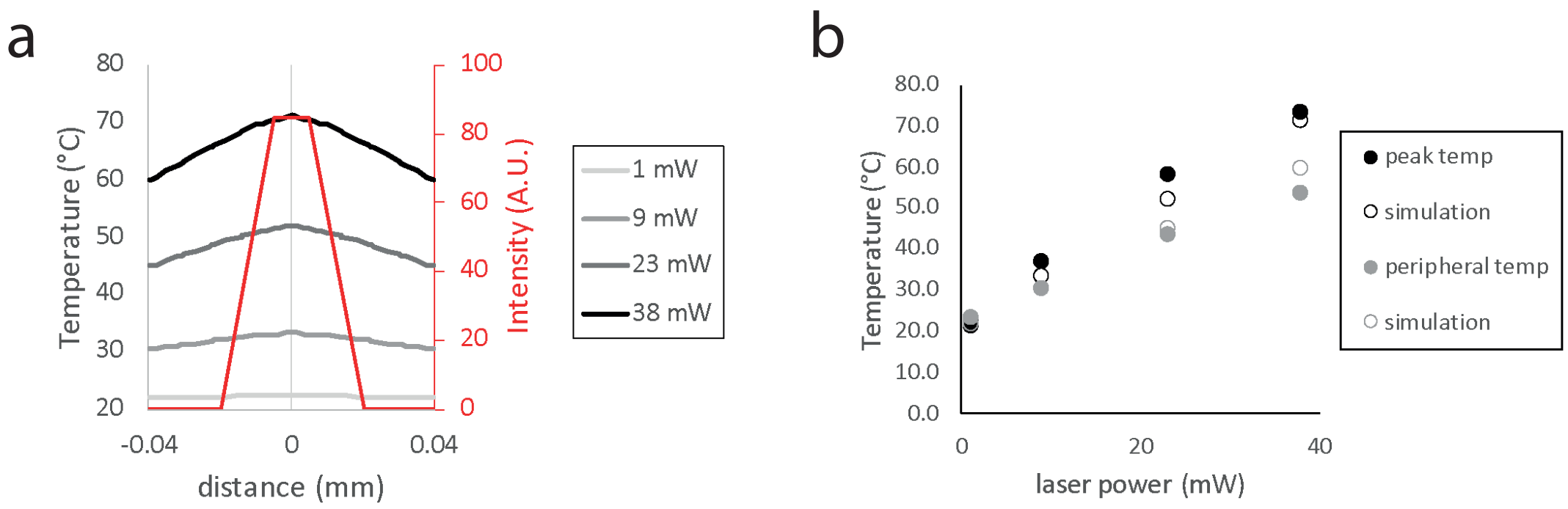

Supplementary Figure 9: Simulation of thermal dissipation in water upon IR-laser irradiation

a) Temperature profiles (left axis) obtained 90 µm within a water sample (surface: 81 mm^2, height: 1.25 mm) upon irradiation by a 1470-nm laser at different laser powers (black and grey lines) with a diameter of 24 µm FWHM (red line, right axis) obtained by finite element simulations. b) comparison of peak temperatures (black circles) and peripheral temperatures (40 µm distance to the center, grey circles) from thermometry by fluorescence lifetime (filled circles, Figure 3c) with finite-element simulations (empty circles)

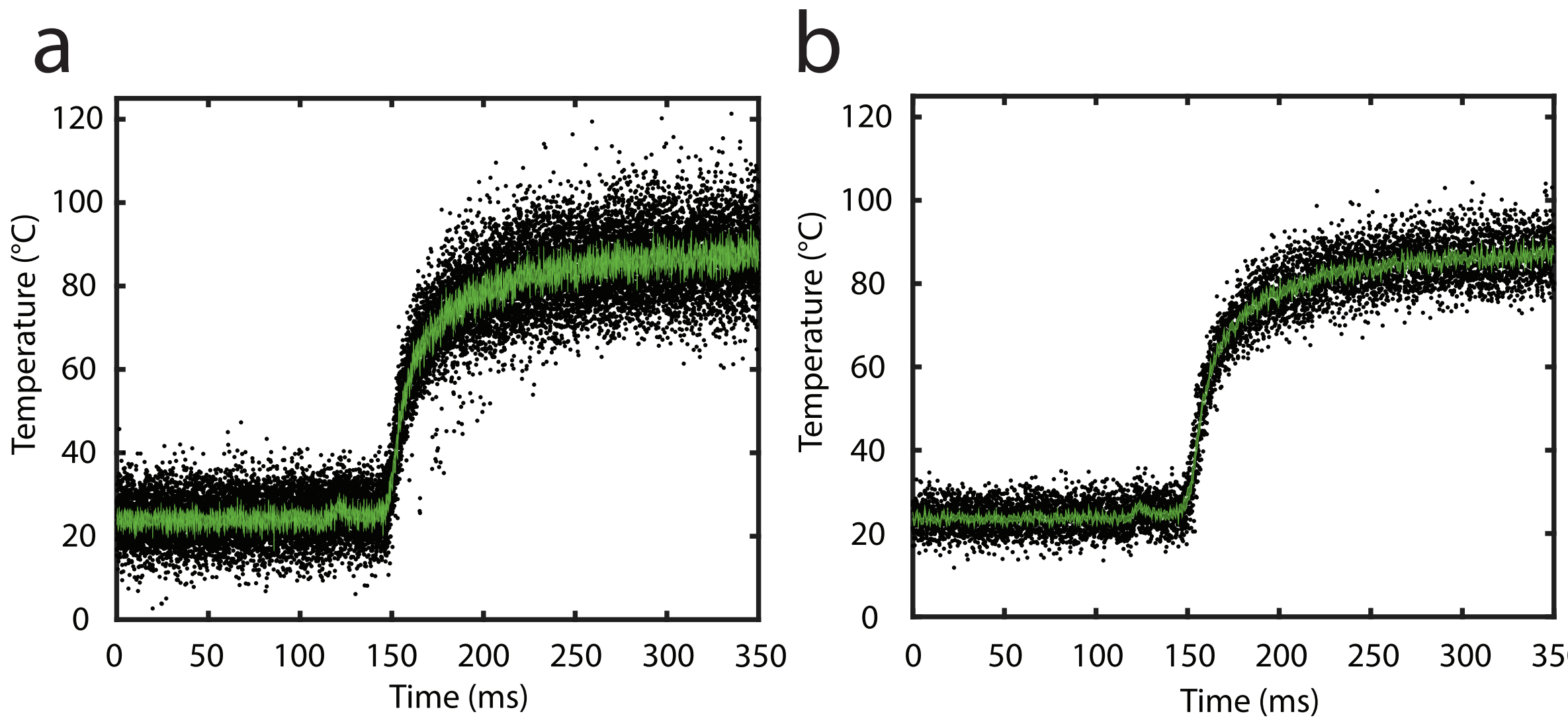

Supplementary Figure 10: Temporal response of infrared laser heating sampled at 200 or 500 µs
Temperature course upon turning-on 1470-nm laser illumination (110 mW), measured in the center of a flat-top laser beam of Ø = 160 µm. Black dots: Accumulated data points from n=21 experiments; green lines: mean ± s.e.m. a) sampling rate: 200 µs; b) 500 µs

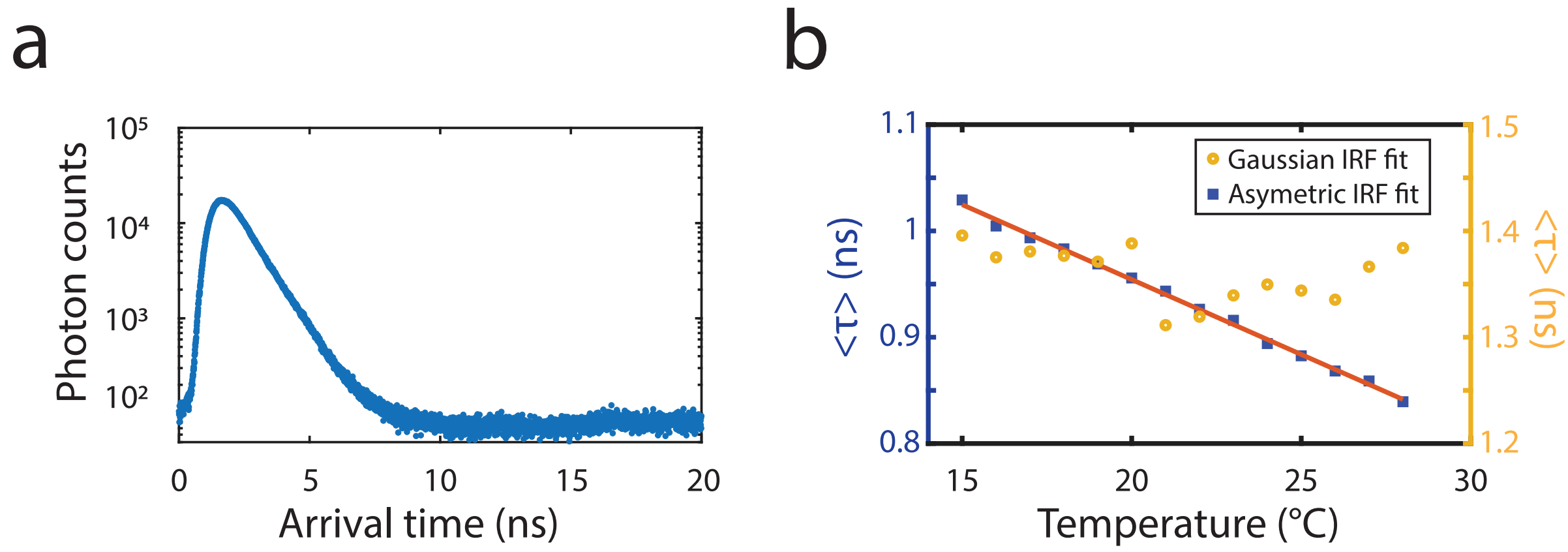

Supplementary Figure 11: Fluorescence of Cy5 induced by a pulsed 775-nm laser

a) Lifetime histogram of 6 µM Cy5 in water excited by a doughnut-shaped 775-nm laser at 23 mW b) Plot of mean excited state lifetime (τ) of 6 µM Cy5 in water obtained by a fit using a gaussian approximation of the IRF (yellow symbols, right axis) or an IRF fitted by an asymmetric 5-parameter function (dark blue symbols, left axis) against temperature (N=2) together with a linear regression of the latter data (red line)

a

	Channel 1<br>415 nm- 460 nm	Channel 2<br>525 nm - 555 nm	Channel 3<br>575 nm -630 nm	Channel 4<br>655 nm - 720 nm
DyLight 405	100	0.003	0.0008	0.0005
mCitrine	0.9	100	0.03	0.0009
mCherry	0.07	0.9	100	0.3
Cy5	0.5	0.007	0.018	100

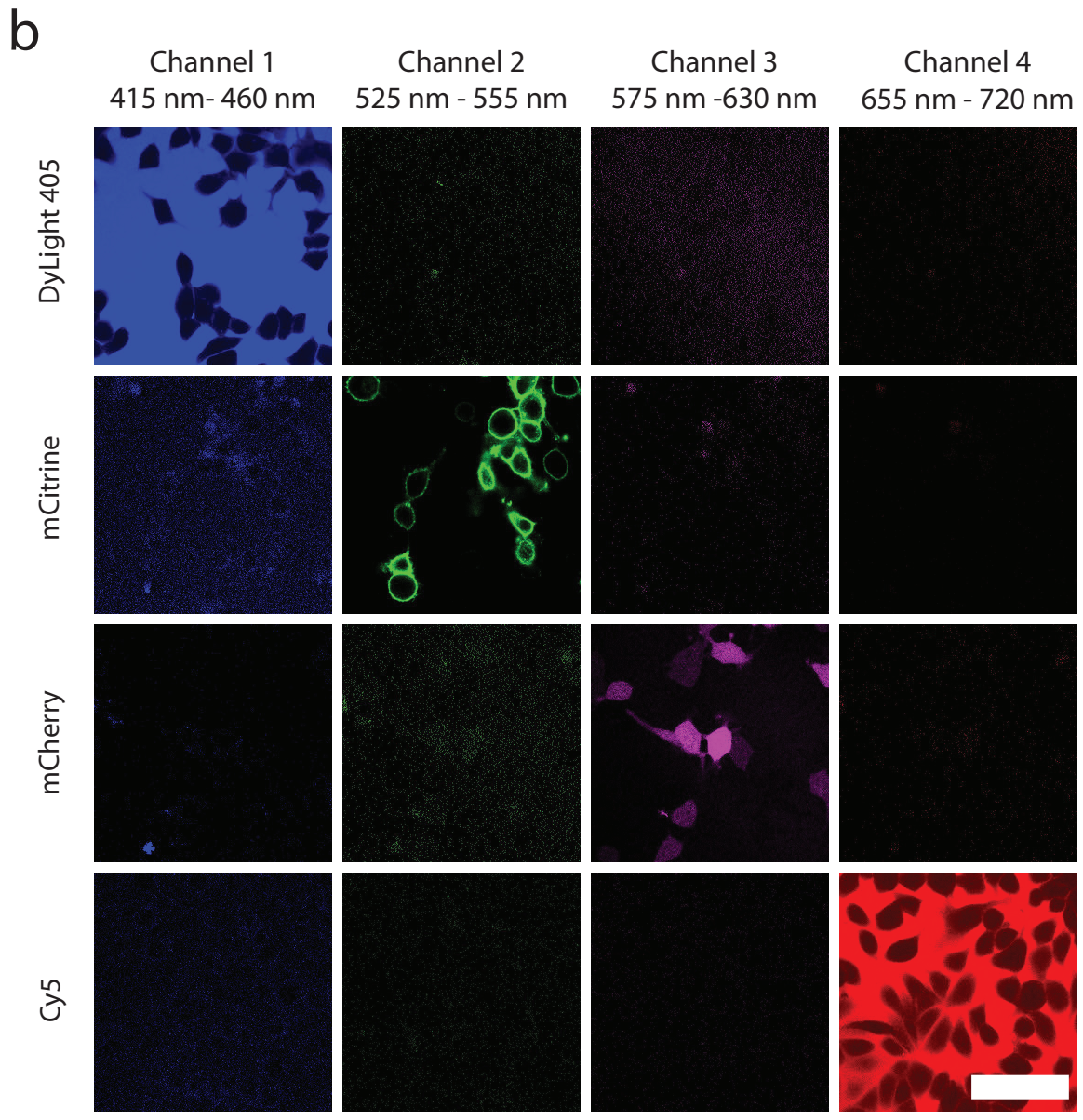

Supplementary figure 12: Fluorescence crosstalk in 4-color fluorescence imaging

a) Quantification of fluorescence signal produced by purified fluorophores or fluorescent proteins in 4 different fluorescent emission channels as indicated. Fluorescent intensities were measured at the same settings as the images in Figure 3a and Suppl. Figure 12b and normalized to the maximum (%). Laser excitation: DyLight 405 (405 nm), mCitrine (488 nm), mCherry (561 nm), Cy5 (640 nm) b) Example images of MCF7 cells acquired with the same settings as in Figure 3a with individual dyes. Contrast was maximized for each image individually to visualize weak signals. Top row: DyLight 405, 2nd row: EGFR-mCitrine, 3rd row: PTB-mCherry, bottom row: Cy5; Scale bar 50 µm

Supplementary Table 1: Fit parameters with 95% confidence intervals

Derived fit parameters for the calibration curve of Cy5 in water together with their 95% confidence interval

	**$a*T+b$**	**$a*e^{bT}$**	**$a*e^{bT}+c*e^{dT}$**	**aT^3+bT^2+cT+d**
**a**	-0.0146 [-0.01469; -0.01456]	1.6002 [1.5943; 1.6060]	1.6991 [1.6659; 1.7323]	$8.1*10^{-7}$ [$7.5*10^{-7}$; $8.6*10^{-7}$]
**b**	1.4146 [1.4132; 1.4160]	-0.0163 [-0.01640; -0.01627]	-0.0171 [-0.0174; -0.0169]	$-3.9*10^{-5}$ [$-4.6*10^{-5}$; $-3.3*10^{-5}$]
**c**			-0.2950 [-0.3270; -0.2630]	-0.0142 [-0.0144; -0.0139]
**d**			-0.0623 [-0.0675; -0.0571]	1.4134 [1.4113; 1.4154]
